# Development and validation of intravoxel incoherent motion diffusion weighted imaging-based model for preoperative distinguishing nuclear grade and survival of clear cell renal cell carcinoma complicated with venous tumor thrombus

**DOI:** 10.1186/s40644-024-00816-2

**Published:** 2024-12-18

**Authors:** Jian Zhao, Honghao Xu, Yonggui Fu, Xiaohui Ding, Meifeng Wang, Cheng Peng, Huanhuan Kang, Huiping Guo, Xu Bai, Shaopeng Zhou, Kan Liu, Lin Li, Xu Zhang, Xin Ma, Xinjiang Wang, Haiyi Wang

**Affiliations:** 1 Department of Radiology, First Medical Center, Chinese PLA General Hospital, No. 28, Fuxing Road, Haidian District, Beijing, 100853 PR China; 2 Department of Radiology, Second Medical Center, Chinese PLA General Hospital, No. 28, Fuxing Road, Haidian District, Beijing, 100853 PR China; 3 Department of Radiology, Sixth Medical Center, Chinese PLA General Hospital, Beijing, 100037 PR China; 4 Department of Pathology, First Medical Center, Chinese PLA General Hospital, Beijing, 100853 PR China; 5 Department of Urology, Chinese PLA General Hospital, Beijing, 100853 PR China; 6 Department of Innovative Medical Research, Hospital Management Institute, Chinese PLA General Hospital, No. 28, Fuxing Road, Haidian District, Beijing, 100853 PR China

**Keywords:** Renal cell carcinoma, Tumor thrombus, Magnetic resonance imaging, Intravoxel incoherent motion, Diffusion-weighted imaging

## Abstract

**Objective:**

To assess the utility of multiparametric MRI and clinical indicators in distinguishing nuclear grade and survival of clear cell renal cell carcinoma (ccRCC) complicated with venous tumor thrombus (VTT).

**Materials and methods:**

This study included 105 and 27 patients in the training and test sets, respectively. Preoperative MRI, including intravoxel incoherent motion diffusion-weighted imaging (IVIM-DWI), was performed. Renal lesions were evaluated for IVIM-DWI metrics and conventional MRI features. All the patients had postoperative histologically proven ccRCC and VTT. An expert uropathologist reviewed all specimens to confirm the nuclear grade of the World Health Organization/ International Society of Urological Pathology (WHO/ISUP) of the tumor. Univariate and multivariable logistic regression analyses were used to select the preoperative imaging features and clinical indicators. The predictive ability of the logistic regression model was assessed using receiver operating characteristic (ROC) analysis. Survival curves were plotted using the Kaplan–Meier method.

**Results:**

High WHO/ISUP nuclear grade was confirmed in 69 of 105 patients (65.7%) in the training set and 19 of 27 patients (70.4%) in the test set, respectively (*P* = 0.647). D_p_ROI_Low_, tumor size, serum albumin, platelet count, and lymphocyte count were independently related to high WHO/ISUP nuclear grade in the training set. The model identified high WHO/ISUP nuclear grade well, with an AUC of 0.817 (95% confidence interval [CI]: 0.735–0.899), a sensitivity of 70.0%, and a specificity of 77.8% in the training set. In the independent test set, the model demonstrated an AUC of 0.766 (95% CI, 0.567–0.966), a sensitivity of 79.0%, and a specificity of 75.0%. Kaplan–Meier analysis showed that the predicted high WHO/ISUP nuclear grade group had poorer progression-free survival than the low WHO/ISUP nuclear grade group in both the training and test sets (*P* = 0.001 and *P* = 0.021).

**Conclusions:**

IVIM-DWI-derived parameters and clinical indicators can be used to differentiate nuclear grades and predict progression-free survival of ccRCC and VTT.

**Supplementary Information:**

The online version contains supplementary material available at 10.1186/s40644-024-00816-2.

## Introduction

Renal cell carcinoma (RCC) accounts for about 3% of all cancers, and the age-standardized incidence rate in Western countries is 10 per 100,000 persons, resulting in 13,920 deaths in the United States in 2022 [1]. Clear cell renal cell carcinoma (ccRCC) is the most common subtype of RCC, characterized by high invasiveness [2]. The incidence rate of venous tumor thrombus (VTT) in RCC is up to 13.3% [3]. The optimal treatment for RCC and VTT is surgical resection to obtain local control in patients without distant metastasis, which may enable more than half of patients to survive in the long term [4]. However, surgery for locally advanced RCC complicated with tumor thrombus was one of the most challenging surgeries in urology, which was complex and expensive, and severe perioperative complications occasionally occurred [5]. The perioperative complications of surgical removal of RCC and inferior vena cava (IVC) tumor thrombus are up to 35.3% [6, 7]. Therefore, patients with ccRCC and VTT who may have poor prognoses may not be suitable for surgical resection first.

Currently, whether patients with RCC and VTT should receive neoadjuvant therapy is controversial. In the recent era of immunotherapy, combination therapy with immuno-oncologic agents and tyrosine kinase inhibitors is the preferred treatment for metastatic RCC [8]. Similar to the treatment strategy for metastatic RCC, surgery may no longer prioritize immunotherapy treatment for RCC and VTT, especially for high-risk patients [9]. The World Health Organization/ International Society of Urological Pathology (WHO/ISUP) nuclear grade is one of the most important prognostic factors for tumor progression in ccRCC. Using the WHO/ISUP nuclear grading system, tumors can be divided into low-grade tumors (grades 1–2, manifested as small and round tumor cells, homogeneous nuclei, and barely perceptible nucleoli) or high-grade tumors (grades 3–4, with large and complex-shaped nuclei, prominent nucleoli, and possibly heavy chromatin clumps) [10]. Multiple studies have confirmed that patients with high WHO/ISUP grading in ccRCC have poorer prognosis after surgery [11–13]. Given that patients with high WHO/ISUP nuclear grade have a worse prognosis and surgery is complex in cases with tumor thrombus, preoperative identification of patients with high WHO/ISUP nuclear grade is of great significance in assisting clinical decision-making.

Due to the high spatiotemporal heterogeneity of ccRCC, a biopsy cannot accurately reflect the entire tumors’ WHO/ISUP nuclear grade [14]. The nuclear-to-cytoplasm ratio increases as the WHO/ISUP nuclear grade of ccRCC increases [15]. Diffusion-weighted imaging measures the free movement ability of water molecules, which is influenced by many factors, including nuclear-to-cytoplasm ratio, cell structure, membrane integrity, and viscosity [14]. Given this, it is reasonable to detect the difference in nuclear characteristics between low-grade and high-grade ccRCC based on the diffusion information of the tumors. Intravoxel incoherent motion diffusion-weighted imaging (IVIM-DWI) is an advanced MRI technology that can distinguish between microcirculation reperfusion and true diffusion of water molecules and can quantitatively or semi-quantitatively reflect the functional status and microstructural characteristics of tissues. IVIM-DWI has been reported to distinguish renal tumor subtype [16], and to evaluate renal allograft function in the early and late phases of transplantation [17], renal interstitial fibrosis [18], and even VTT consistency of RCC [19]. However, whether IVIM-DWI-derived parameters could predict the WHO/ISUP nuclear grade of ccRCC and VTT has not been investigated so far. Besides, preoperative clinical laboratory indicators are low-cost, convenient, noninvasive, and rapidly obtained indexes, and are potential predictive factors related to tumor invasion and prognosis [20]. However, whether clinical laboratory indicators are associated with the WHO/ISUP nuclear grade of ccRCC and VTT has not been reported.

Thus, our study aims to evaluate the value of preoperative MRI indicators (especially IVIM-DWI) and clinical parameters in determining the WHO/ISUP nuclear grade of RCC and VTT, and differentiating the prognosis of patients.

## Materials and methods

This study was conducted following the Helsinki Declaration.

### Patients and study cohorts

**Fig. 1:**
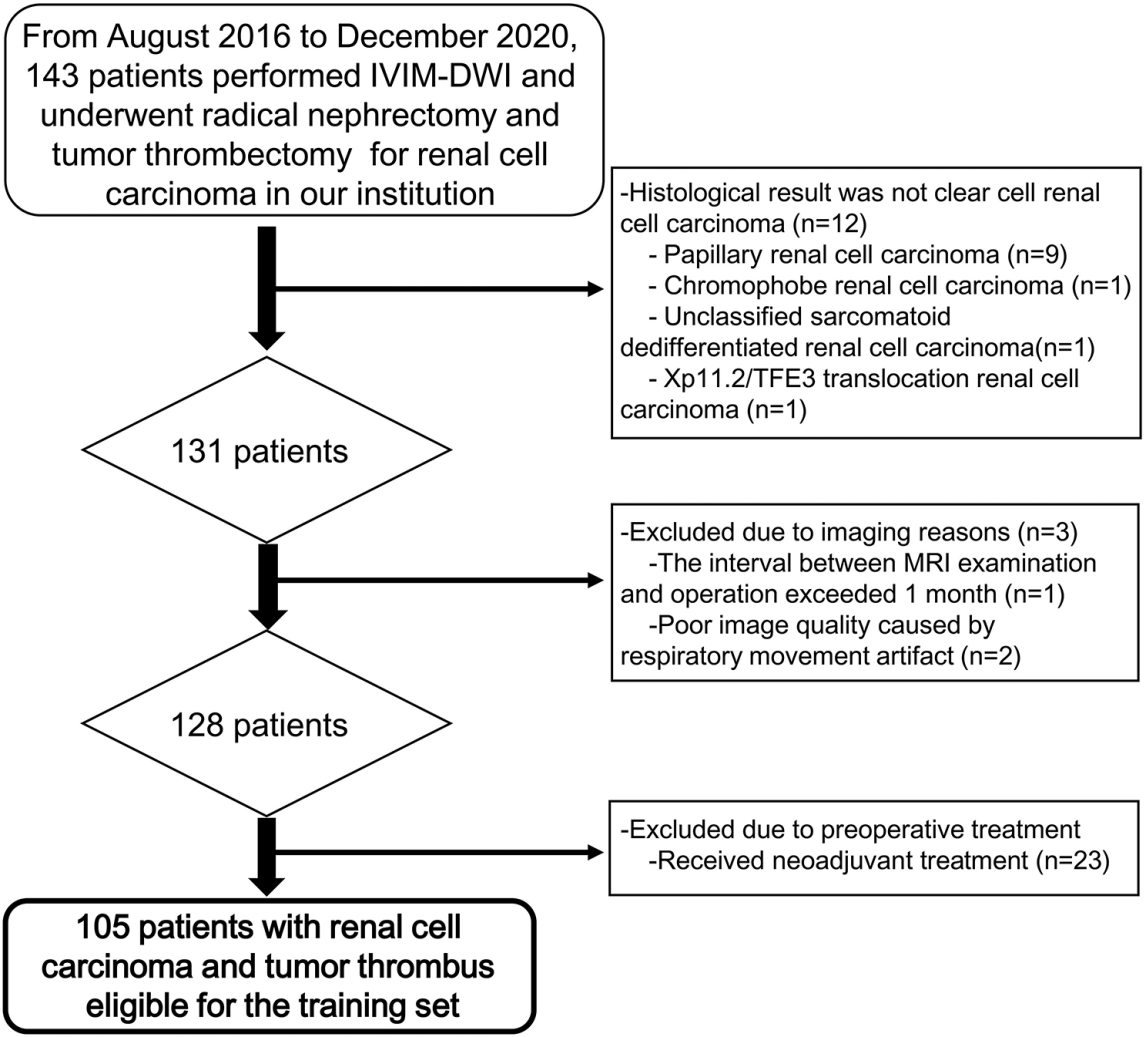
Workflow of patient selection for the training set

Consecutive patients who underwent radical nephrectomy and tumor thrombectomy for RCC from August 2016 to December 2020 in Chinese PLA General Hospital were retrospectively collected to construct the model. Exclusion criteria were (a) malignant neoplasms other than ccRCC in pathology; (b) the interval between preoperative MRI examination and operation exceeds 30 days; (c) unsatisfactory quality of images due to respiratory motion artifacts; and (d) accepted neoadjuvant therapy. Finally, 105 patients were included in the training set. A flowchart of patient selection is provided in Fig. 1. Twenty-seven patients who underwent radical nephrectomy and tumor thrombectomy for ccRCC and VTT from January 2021 to January 2022 were prospectively collected and enrolled into the test set.

### MRI protocol

All MRI examinations were performed on a 3.0-T system (Discovery MR750, GE Healthcare, Milwaukee, Wisconsin, USA) using an eight-channel phased-array abdominal coil. Examinations included our institution’s unenhanced renal mass protocol, contrast-enhanced MRI (CE-MRI), and IVIM-DWI. CE-MRI was performed after administration of gadobenate dimeglumine (0.1 mmol/kg of body weight; Bracco) at a rate of 1.5 mL/s, followed by a 20-mL saline flush. Contrast-enhanced images were acquired during multiple phases, including the corticomedullary phase (CMP), nephrographic phase (NP), and delayed phase at 25–30 s, 60–70 s, and 240 s after contrast material administration. MRI acquisition parameters are summarized in Table S1 (online). The IVIM-DWI images were acquired through fat-suppressed single-shot echo planar imaging sequence in the axial plane with free-breathing, and *b* values were 0, 20, 50, 100, 150, 200, 400, 600, 800 s/mm^2^ in three orthogonal motion-probing gradients. The number of excitations (NEX) for each b was 1, 2, 2, 2, 2, 2, 4, 4, and 4, respectively. The remaining parameters were as follows: TR, 3500 msec; TE, 58 msec; matrix, 160 × 160; FOV, 38 cm × 38 cm; receiver bandwidth, 110 kHz; acceleration factor, two; thickness, 5.0 mm; slice gap, 1 mm; the number of slices, 30; acquisition time, 4:04 min.

### Pre-procession of images

The acquired IVIM-DWI images were processed on Matlab R2013a (Mathworks, Natick, MA). Mono- and bi-exponential models were generated from multiple-*b*-value DWI data on a voxel-by-voxel basis. The calculation code is listed in Supplemental Content (online). Bi-exponential model (IVIM model) followed the Levenberg-Marquardt fit for nonlinear fitting, and the formula was as follows:

1 $$S(b) = S(0) [f \times e^{-bDp} + (1-f) \times e^{-bDt}]$$

Where *S(b)* was the mean signal intensity on DWI under the given *b* value, *S(0)* was the mean signal intensity on DWI under the *b* value of 0 s/mm^2^. Firstly, the D_t_ value was calculated by linear least squares by using the logarithm of signal intensity at *b*-values of 400 and 800 s/mm^2^. Secondly, *S(0)*, D_p_, and *f* were estimated from a bi-exponential fit with a fixed D_t_ value (obtained above) using all *b*-values. D_t_ was the true diffusion coefficient, D_p_ was the pseudo-diffusion coefficient, and *f* was the perfusion fraction linked to the microcirculation [21].

Quantitative ADC maps were calculated based on a mono-exponential leastsquares fit with a voxel-by-voxel basis using the entire range of *b*-values [22]. The formula was as follows:

2 $$ln (S_b/S_0) = -b \times ADC$$

Where *S*_*b*_ represented the mean signal intensity with diffusion weighting *b* and *S*_*0*_ corresponded to signal intensity for *b* value of 0 s/mm^2^. *S*_*0*_ and ADC values were simultaneously estimated from a mono-exponential fit.

### Lesion segmentation

**Fig. 2:**
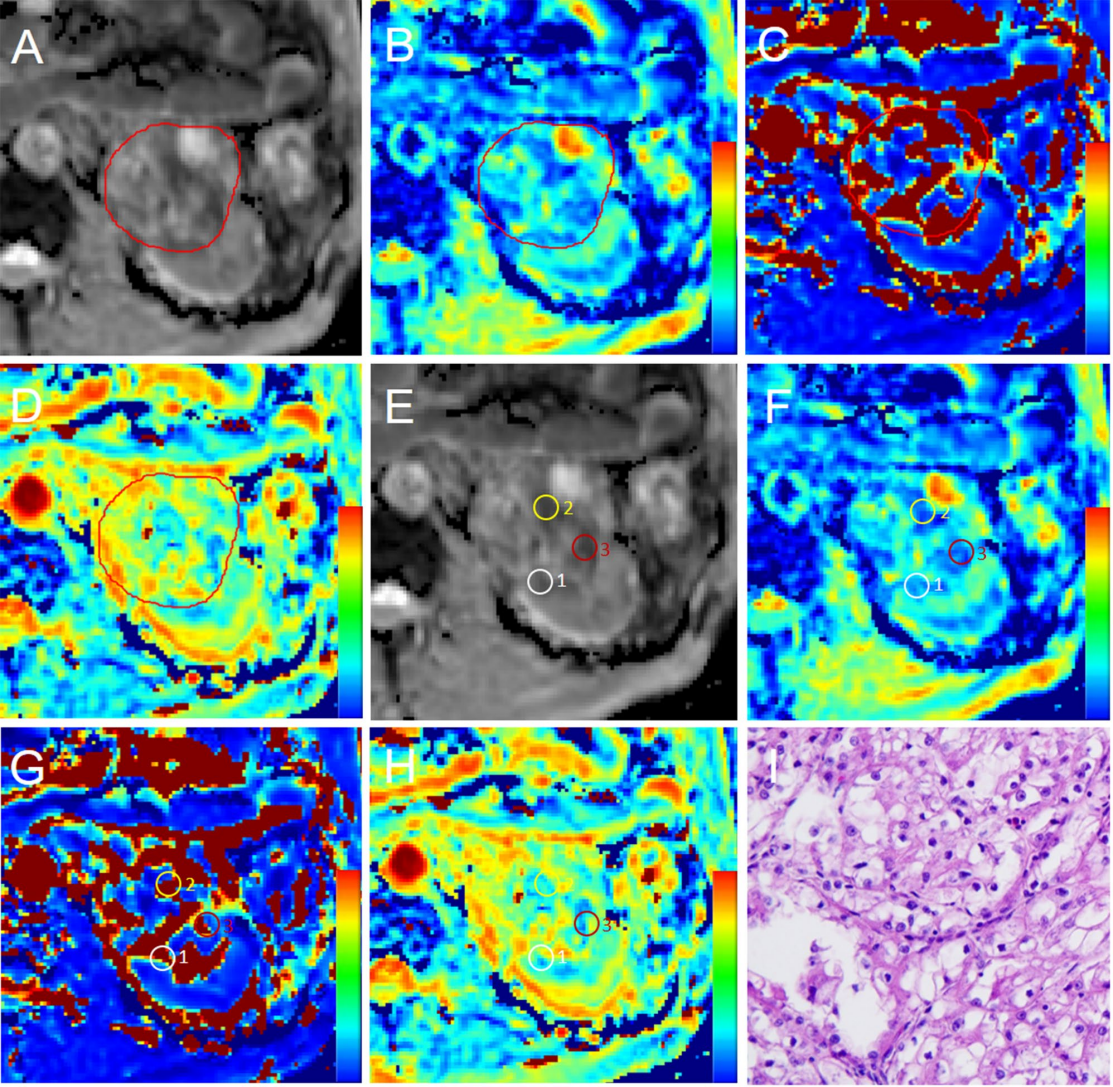
A 67-year-old male patient with a left-sided 6.6‑cm low WHO/ISUP nuclear grade ccRCC and tumor thrombus. Free-hand regions of interest (ROIs) were placed on the largest section of the tumor on ADC (**A**), copied to D_t_ (**B**), D_p_ (**C**), and f maps (**D**). ROIs with a fixed area of 50 mm^2^ were delineated at the lowest ADC areas of the tumor on ADC (**E**), copied to Dt (**F**), Dp (**G**), and f maps (**H**). A small white circle represents ROI1, yellow represents ROI2, and red represents ROI3. (**I**) Pathological analysis confirmed low-grade ccRCC (WHO/ISUP grade 2) (hematoxylin and eosin, × 400)

**Fig. 3:**
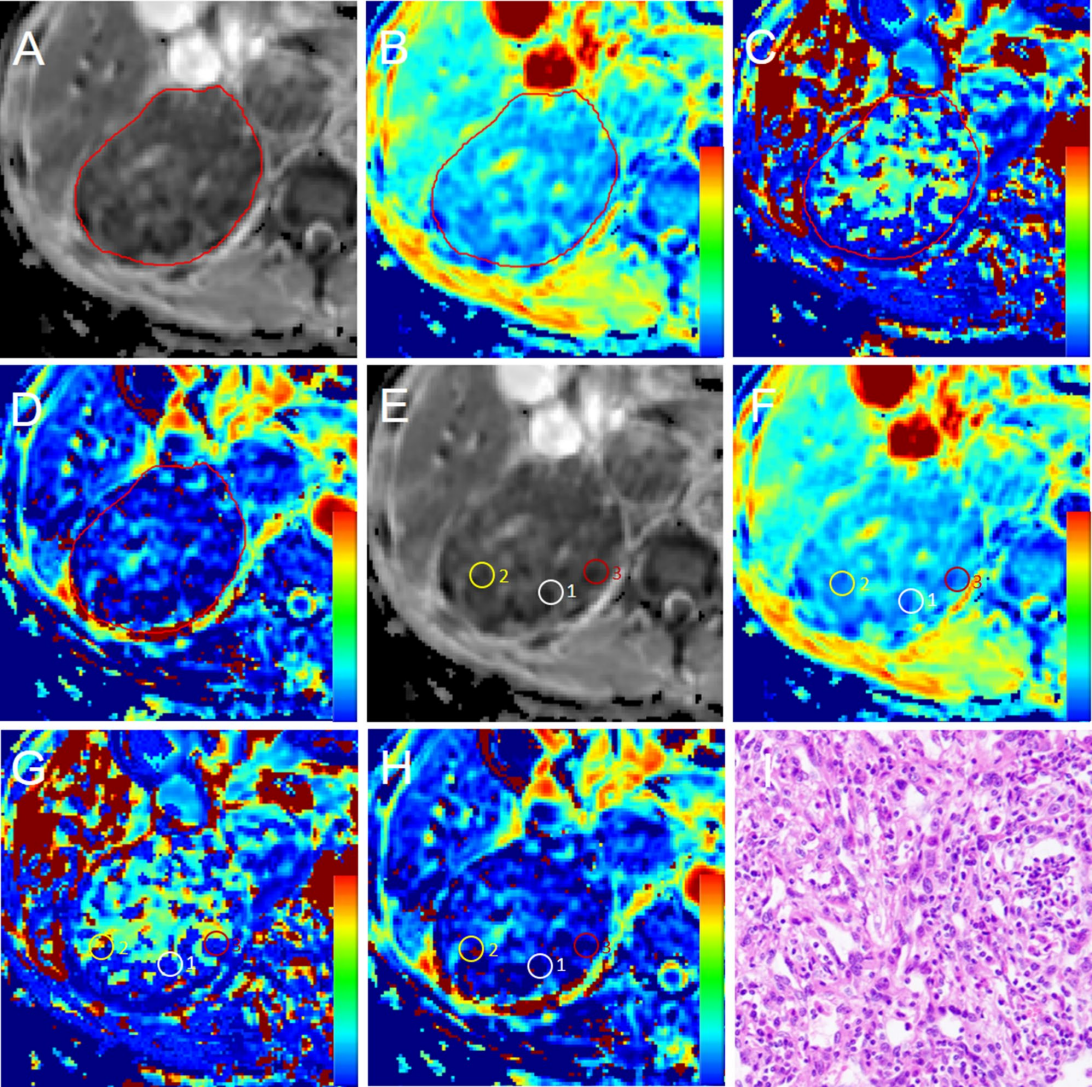
A 62-year-old male patient with a right-sided 15.7‑cm high WHO/ISUP nuclear grade ccRCC and tumor thrombus. Free-hand regions of interest (ROIs) were placed on the largest section of the tumor on ADC(**A**), D_t_(**B**), D_p_(**C**), and f maps(**D**). ROIs with a fixed area of 50 mm^2^ were delineated at the lowest ADC areas of the tumor on ADC(**E**), copied to D_t_(**F**), D_p_(**G**), and f maps(**H**). A small white circle represents ROI1, yellow represents ROI2, and red represents ROI3. (**I**) Pathological analysis (hematoxylin and eosin, × 400) confirmed high-grade ccRCC (WHO/ISUP grade 4)

After pre-processing of IVIM-DWI images, two board-certified radiologists (reader1 [JZ] and reader2 [MW], with 15 and 5 years of experience in genitourinary imaging, respectively) independently drew regions of interest (ROIs) in ITK-SNAP software (version 3.6.0, open-source software; https://itk.org/), blinding to patients’ histopathological information. ROIs were drawn manually through two methods. The first method was to draw ROIs on the largest section of the primary tumor on the 2D apparent diffusion coefficient (ADC) maps, ensuring that ROIs included the primary tumor area as large as possible (defined as ROI__largest_) (Figs. 2A-D and 3A-D). Furthermore, the adjacent lower and upper sections were also selected to draw ROIs in the same method. The averaged values of the three sections were computed. The second method was to draw three ROIs where the lowest ADC value was visualized in the tumor (defined as ROI__low_) (Figs. 2E-H and 3E-H). The three ROI__low_ were drawn with a fixed area of 50 mm^2^. The averaged values of the three ROI__low_ were computed. The ROI on the ADC map was copied to D_t_, D_p,_ and *f* maps. Only a few tumors (*n* = 4) have doubts when delineating the area of interest. In these cases, the two radiologists consulted and resolved the issue. The averaged values of each parameter generated by the two radiologists were used for the final analysis.

### Analysis of conventional imaging sign

**Fig. 4:**
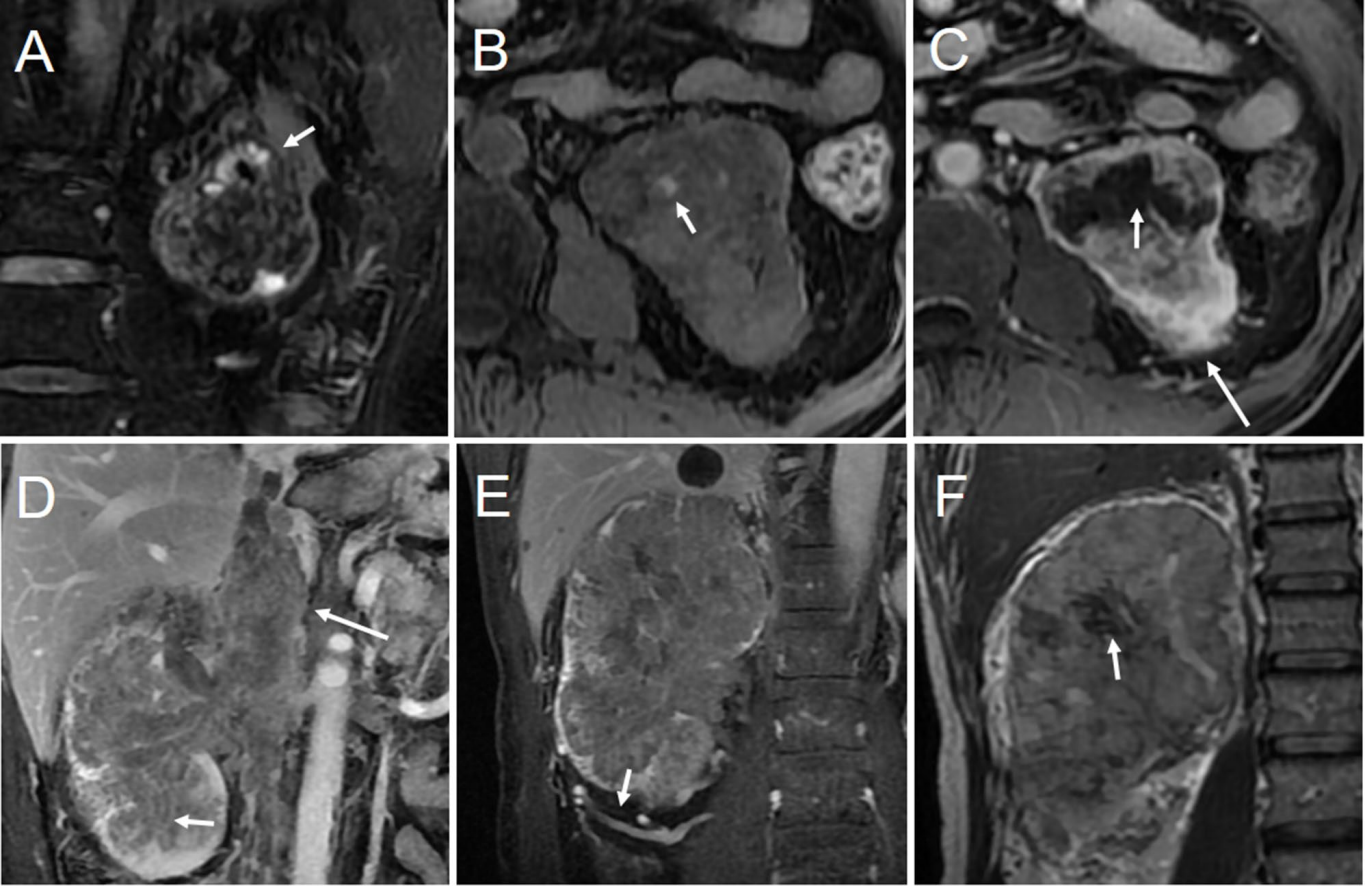
Conventional imaging signs. (**A**) T2‑weighted axial image displayed pseudo capsule (short arrow). (**B**) Hyper-intensity on fat-suppressed T1-weighted imaging indicated hemorrhage (short arrow). (**C**) Post‑contrast T1‑weighted image showed non‑enhancing necrosis (short arrow); The tumor had an ill-defined margin with perirenal fat, which means that the perirenal fat was invaded (long arrow). (**D**) The tumor showed infiltrating growth (short arrow), and the tumor thrombus grew into the inferior vena cava (long arrow). (**E**) Post‑contrast T1‑weighted image showed peritumoral neovascularization (short arrow). (**F**) T2‑weighted coronal image displayed T_2_ low-intensity areas (T2LIA) (short arrow). The top row of images is the same patient in Fig. 2, whereas the following row of images belongs to the patient in Fig. 3

Two board-certified radiologists (reader1, JZ; reader2, MW) independently assessed conventional imaging signs of images on RadiAnt software (version 2021.2) and were blinded to patients’ histopathological information. The following features were recorded: tumor size (defined as the largest diameter of the tumor in any dimension), pseudo-capsule (defined as the presence of a complete hypo-intense ring around the tumor on T2-weighted imaging [T_2_WI], Fig. 4A) [23], hemorrhage (defined as hyper-intensity on fat-suppressed T1-weighted imaging [T_1_WI]) and no enhancement, Fig. 4B) [24], necrosis (defined as irregular high T_2_WI area in the tumor, without enhancement, Fig. 4C) [25]; infiltrative growth (defined as the boundary between the tumor and renal parenchyma was unclear, Fig. 4D) [26], peritumoral neovascularization (defined as asymmetrically dilated irregular vessels within the Gerota fascia adjacent to the affected kidney, Fig. 4E) [27], T_2_ low-intensity areas (T2LIA, defined as multiple > 1 cm T_2_ low-intensity areas or had > 3 cm hypo-intensity areas on T_2_WI, Fig. 4F), which was referred to Takeuchi’s criteria [28], perirenal fat invasion (defined as an exophytic component of the lesion with an ill-defined margin between tumor and perirenal fat, Fig. 4C) [29], tumor thrombus level (according to Mayo classification) [30], lymph node metastasis (defined as the short diameter of lymph nodes > 1 cm or with suspicious characteristics, such as rounded appearance, heterogeneity or irregular shape) [31]. After conducting an independent evaluation, the two radiologists discussed all cases to reach in consensus. For disagreed cases, the third senior board-certified radiologist (HW, with 24 years of experience in urogenital diagnosis) made the final decision. The final consensual result was used for analysis.

### Collection of clinical data

The following variables were collected through the electronic medical record system: age, gender, clinical symptoms, body mass index (BMI), hypertension, diabetes, drinking history, smoking history, and postoperative adjuvant therapy. The laboratory indicators included white blood cell (WBC) in urine, red blood cell (RBC) in urine, proteinuria, serum albumin, total protein, hemoglobin, alkaline phosphatase, lactate dehydrogenase, blood glucose, serum urea, serum creatinine, serum calcium, neutrophil count, lymphocyte count, and platelet count. Only < 1% of the values have missing values, which are replaced by the median.

### Surgical histopathologic analyses

Histopathologic analyses focused on the primary tumor of RCC. The primary tumor specimens were consecutively sampled every 3–4 mm and stained with hematoxylin and eosin. A genitourinary pathologist (XD, 26 years of experience in genitourinary pathology) blindly reviewed all specimens and confirmed the WHO/ISUP nuclear grade, T stage, and N stage. The WHO/ISUP nuclear grade was based on the 2012 International Society of Urological Pathology Consensus [32]. The WHO/ISUP nuclear grade was dichotomized: (a) low grade: grades 1 and 2; (b) high grade: grades 3 and 4. Microscopic manifestations of representative cases with low-grade and high-grade ccRCC are shown in Figs. 2I and 3I.

### Followed-up and clinical endpoint

After the surgery, all patients were followed up every 3–6 months for the first 3 years, and every 6–12 months thereafter, until the patient’s death. Follow-up was focused on blood tumor markers and imaging examination of the chest and abdomen. The follow-up time of patients is calculated from the date of pathological diagnosis to the last contact or death date. Progression-free survival (PFS) refers to the time from the date of surgery to recurrence or death from the disease, whichever occurs first. All recurrent diagnoses were confirmed by biopsy and/or imaging follow-up evidence. All follow-ups ended in December 2023.

### Logistic regression analysis

Employing the maximum Youden index (defined as sensitivity + specificity − 1) in the training set, we convert continuous variables into binary variables for further analysis. Imaging features and clinical parameters were explored by using univariable logistic regression analysis to determine the potential factors related to the WHO/ISUP nuclear grade. Variables that were potentially related to WHO/ISUP nuclear grade (*P* < 0.05) in the univariate logistic regression analysis were included in the multivariable logistic regression model (backward LR) to further evaluate their association with the WHO/ISUP nuclear grade. During the multivariable logistic regression analysis, the WHO/ISUP nuclear grade (defined as 1 for high grading and 0 for low grading) acted as the dependent variable, and preoperative clinical, laboratory, and imaging indicators as independent variables.

### Statistical methods

Statistical analysis was performed using R software (4.2.2 Version, R Foundation for Statistical Computing, Vienna, Austria) and MedCalc (version 20). The normality of data distributions was accessed by the Shapiro-Wilk test. Continuous variables were described as mean and standard deviation (SD) or the median and interquartile ranges (IQRs) according to the normality of data distributions, and categorical variables were expressed as frequency and proportion. Student’s *t*-test or Mann-Whitney *U* test was used to compare the differences of continuous variables between groups, and Fisher’s exact tests or chi-squared tests were used for categorical variables. The Cohen kappa coefficients assessed the agreement of the subjective imaging features, and the intraclass correlation coefficient (ICC) was employed to determine the agreement of continuous parameters measured by the two independent radiologists. Kappa values and ICC were interpreted as follows: 0.001-0.200 indicates slight consistency; 0.201-0.400 indicates fair consistency; 0.401-0.600 indicates moderate consistency; 0.601 -0.800 indicates substantial consistency; 0.801-1.000 indicates excellent agreement. The threshold of each continuous variable was obtained by the maximal Youden index. The diagnostic performance of independent indicators and the logistic regression model was assessed by the receiver operating characteristic (ROC) curve and associated areas under the ROC curve (AUCs), sensitivity, specificity, positive predictive value, and the negative predictive value corresponding to the threshold, respectively. Differences in AUCs were compared by DeLong’s test. Survival curves were plotted using the Kaplan–Meier method, and the log-rank test was used to compare the differences. All reported *P* values were two-tailed and *P* < 0.05 was considered statistically significant.

## Results

### Population characteristics

The final sample comprised 105 patients in the training set (mean age, 57.5 years ± 10.1 [SD]) and 27 patients in the test set (mean age, 56.6 years ± 11.2 [SD]) (*P* = 0.664). Characteristics of patients in the training set and the test set are presented in Table 1. High WHO/ISUP nuclear grade was diagnosed in 69 of 105 patients (65.7%) in the training set and 19 of 27 patients (70.4%) in the test set, respectively (*P* = 0.647). The baseline clinical characteristics and histopathologic findings did not differ between training and test sets (range, *P* = 0.056–0.878).

**Table 1:** Comparison of baseline clinical characteristics of patients in the training and test sets

Characteristics	Training set (*n* = 105)	Test set (*n* = 27)	*P* value
Age (years, mean [SD]) *	57.52 (10.10)	56.56 (11.16)	0.664
Gender			0.145
Male	75 (71.4)	23 (85.2)	
Female	30 (28.6)	4 (14.8)	
Side			0.056
Right	65 (61.9)	22 (81.5)	
Left	40 (38.1)	5 (18.5)	
BMI (kg/m^2^, mean [SD]) *	24.65 (3.29)	24.32 (3.47)	0.642
Clinical symptoms			0.428
With	27 (25.7)	9 (33.3)	
Without	78 (74.3)	18 (66.7)	
Diabetes			0.540
With	84 (80.0)	23 (85.2)	
Without	21 (20.0)	4 (14.8)	
Hypertension			0.821
With	57 (54.3)	14 (51.9)	
Without	48 (45.7)	13 (48.1)	
Smoking history			0.322
With	54 (51.4)	11 (40.7)	
Without	51 (48.6)	16 (59.3)	
Drinking history			0.064
With	52 (49.5)	8 (29.6)	
Without	53 (50.5)	19 (70.4)	
Mayo classification			0.878
0	35 (33.3)	9 (33.3)	
I	7 (6.7)	3 (11.1)	
II	41 (39.0)	11 (40.7)	
III	8 (7.6)	2 (7.4)	
IV	14 (13.3)	2 (7.4)	
Tumor size (cm, median [IQR]) *	7.90 (6.40, 9.70)	9.40 (6.35, 11.70)	0.171
T stage			0.160
T3a	37 (35.2)	12 (44.4)	
T3b	34 (32.4)	6 (22.2)	
T3c	34 (32.4)	8 (29.6)	
T4	0 (0.0)	1 (3.7)	
N stage			0.134
N0	96 (91.4)	22 (81.5)	
N1	9 (8.6)	5 (18.5)	
Invasion of IVC wall			0.350
Without	72 (68.6)	21 (77.8)	
With	33 (31.4)	6 (22.2)	
Sarcomatoid differentiation			0.584
Without	90 (85.7)	22 (81.5)	
With	15 (14.3)	5 (18.5)	
Perirenal fat invasion			0.445
Without	87 (82.9)	24 (88.9)	
With	18 (17.1)	3 (11.1)	
Renal sinus fat invasion			0.281
Without	21 (20.0)	8 (29.6)	
With	84 (80.0)	19 (70.4)	
ISUP nuclear grade			0.647
1–2	36 (34.3)	8 (29.6)	
3–4	69 (65.7)	19 (70.4)	
Postoperative adjuvant therapy			0.206
Without	64 (61.0)	20 (74.1)	
With	41 (39.0)	7 (25.9)	

Note Unless otherwise specified, data are numbers of patients, with percentages in parentheses.

* Data are means ± SDs or median and IQRs

### Comparison of clinical and imaging features between patients with low and high WHO/ISUP nuclear grade groups in the training set

We first convert each continuous variable into a binary variable using the maximum Youden index. The maximum Youden index and related parameters corresponding to each continuous variable are shown in Table S2. Among clinical features, clinical symptoms, alkaline phosphatase, serum urea, hemoglobin, serum albumin, aspartate aminotransferase, alanine aminotransferase, creatine kinase, RBC count, lymphocyte count, platelet count, RBC in urine were significantly different between low and high WHO/ISUP nuclear grade groups in the training set (Table 2). Other clinical features were not significantly different, such as BMI, serum creatinine, serum calcium, etc. (Table S3).

**Table 2:** Comparison of clinical features between patients with low and high ISUP nuclear grade clear cell renal cell carcinoma in the training set

Parameter	Low-grade (*n* = 36)	High-grade (*n* = 69)	*P* value
Gender			0.558
Male	27 (75.0)	48 (69.6)	
Female	9 (25.0)	21 (30.4)	
Age			0.934
< 60 years	19 (52.8)	37 (53.6)	
≥ 60 years	17 (47.2)	32 (46.4)	
Side			0.164
Right	19 (52.8)	46 (66.7)	
Left	17 (47.2)	23 (33.3)	
Clinical symptoms			0.007
Without	15 (41.7)	12 (17.4)	
With	21 (58.3)	57 (82.6)	
Alkaline phosphatase			0.045
< 104.1 U/L	31 (86.1)	47 (68.1)	
≥ 104.1 U/L	5 (13.9)	22 (31.9)	
Serum urea			0.002
< 4.52 mmol/L	4 (11.1)	28 (40.6)	
≥ 4.52 mmol/L	32 (88.9)	41 (59.4)	
Hemoglobin			< 0.001
< 121 g/L	9 (25.0)	42 (60.9)	
≥ 121 g/L	27 (75.0)	27 (39.1)	
Serum albumin			0.001
< 42.6 g/L	19 (52.8)	58 (84.1)	
≥ 42.6 g/L	17 (47.2)	11 (15.9)	
Aspartate aminotransferase			0.010
< 13.6 U/L	7 (19.4)	31 (44.9)	
≥ 13.6 U/L	29 (80.6)	38 (55.1)	
Alanine aminotransferase			0.019
< 17.7 U/L	16 (44.4)	47 (68.1)	
≥ 17.7 U/L	20 (55.6)	22 (31.9)	
Creatine kinase			0.003
< 42 U/L	9 (25.0)	38 (55.1)	
≥ 42 U/L	27 (75.0)	31 (44.9)	
RBC count			0.015
< 4.57 × 10^12^/L	20 (55.6)	54 (78.3)	
≥ 4.57 × 10^12^/L	16 (44.4)	15 (21.7)	
Lymphocyte count			0.007
< 1.39 × 10^9^/L	7 (19.4)	32 (46.4)	
≥ 1.39 × 10^9^/L	29 (80.6)	37 (53.6)	
Platelet count			0.001
< 178 × 10^9^/L	15 (41.7)	9 (13.0)	
≥ 178 × 10^9^/L	21 (58.3)	60 (87.0)	
RBC in urine			0.047
Negative	23 (63.9)	30 (43.5)	
Positive	13 (36.1)	39 (56.5)	

Note Data are numbers of patients, with percentages in parentheses

A comparison of imaging features between low and high WHO/ISUP nuclear grade groups in the training set is shown in Table 3. D_p_____ROI_Low_, D_p_ROI_____largest_, D_t_____ROI_Low_, D_t_____ROI_____largest_, *f*____ROI_Low_, *f*____ROI_____largest_, ADC____ROI_Low_, ADC____ROI_____largest_, tumor size, T2LIA, peritumoral neovascularization, necrosis, and Mayo classification were significantly different between low and high WHO/ISUP nuclear grade groups.

**Table 3:** Comparison of imaging features between patients with low and high ISUP nuclear grade clear cell renal cell carcinoma in the training set

Parameter	Low-grade (*n* = 36)	High-grade (*n* = 69)	*P* value
D_p_____ROI_Low_			0.016
< 13.677 × 10^− 3^ mm^2^/s	5 (13.9)	25 (36.2)	
≥ 13.677 × 10^− 3^ mm^2^/s	31 (86.1)	44 (63.8)	
D_p_ROI_____largest_			0.032
< 22.598 × 10^− 3^ mm^2^/s	14 (38.9)	42 (60.9)	
≥ 22.598 × 10^− 3^ mm^2^/s	22 (61.1)	27 (39.1)	
D_t_____ROI_Low_			0.014
< 0.925 × 10^− 3^ mm^2^/s	18 (50.0)	51 (73.9)	
≥ 0.925 × 10^− 3^ mm^2^/s	18 (50.0)	18 (26.1)	
D_t_____ROI_____largest_			0.048
< 1.287 × 10^− 3^ mm^2^/s	12 (33.3)	37 (53.6)	
≥ 1.287 × 10^− 3^ mm^2^/s	24 (66.7)	32 (46.4)	
*f*____ROI_Low_ (×100%)			0.024
< 0.262	11 (30.6)	37 (53.6)	
≥ 0.262	25 (69.4)	32 (46.4)	
*f*____ROI_____largest_ (×100%)			0.029
< 0.382	19 (52.8)	51 (73.9)	
≥ 0.382	17 (47.2)	18 (26.1)	
ADC____ROI_Low_			< 0.001
< 1.135 × 10^− 3^ mm^2^/s	11 (30.6)	49 (71.0)	
≥ 1.135 × 10^− 3^ mm^2^/s	25 (69.4)	20 (29.0)	
ADC____ROI_____largest_			0.003
< 1.824 × 10^− 3^ mm^2^/s	18 (50.0)	54 (78.3)	
≥ 1.824 × 10^− 3^ mm^2^/s	18 (50.0)	15 (21.7)	
Tumor size			0.044
< 6.7 cm	17 (47.2)	19 (27.5)	
≥ 6.7 cm	19 (52.8)	50 (72.5)	
Infiltrative growth			0.249
Without	22 (61.1)	34 (49.3)	
With	14 (38.9)	35 (50.7)	
Pseudocapsule			0.812
Without	20 (55.6)	40 (58.0)	
With	16 (44.4)	29 (42.0)	
T2LIA			0.033
Without	18 (50.0)	20 (29.0)	
With	18 (50.0)	49 (71.0)	
Perirenal fat invasion			0.080
Without	20 (55.6)	26 (37.7)	
With	16 (44.4)	43 (62.3)	
Peritumoral neovascularization			0.041
Without	12 (33.3)	11 (15.9)	
With	24 (66.7)	58 (84.1)	
Hemorrhage			0.656
Without	23 (63.9)	41 (59.4)	
With	13 (36.1)	28 (40.6)	
Necrosis			0.049
With	15 (41.7)	16 (23.2)	
Without	21 (58.3)	53 (76.8)	
Lymph node metastasis			0.126 ^a^
N0	35 (97.2)	61 (88.4)	
N1	1 (2.8)	8 (11.6)	
Mayo classification			0.028
0	13 (36.1)	22 (31.9)	
I	0 (0.0)	7 (10.1)	
II	18 (50.0)	23 (33.3)	
III	4 (11.1)	4 (5.8)	
IV	1 (2.8)	13 (18.8)	

Note Data are numbers of patients, with percentages in parentheses. T2LIA = T_2_ low-intensity areas. ^a^ Comparison was based on Fisher’s exact test

### Logistic regression analysis

**Fig. 5:**
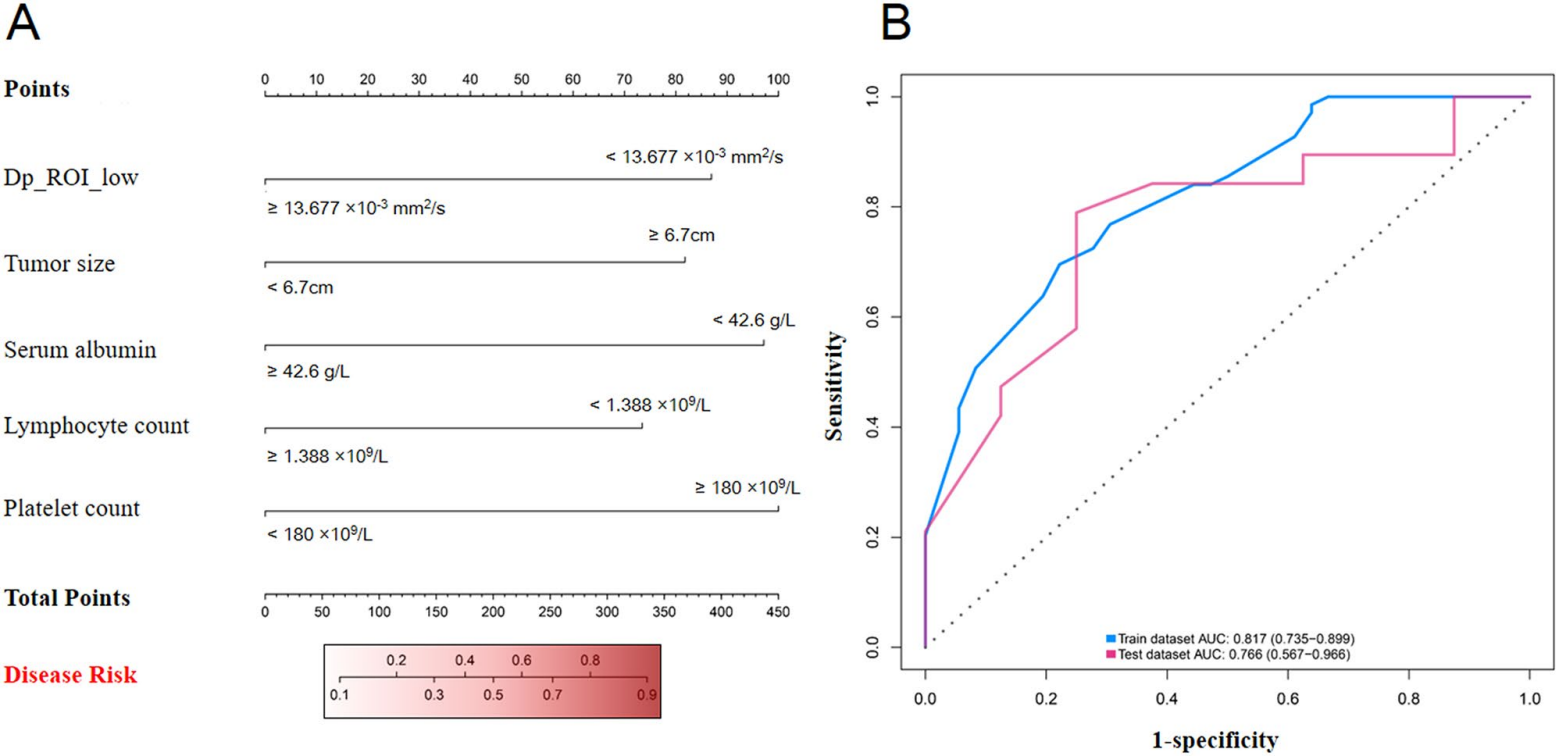
(**A**) Nomogram model for predicting high WHO/ISUP nuclear grade ccRCC and tumor thrombus. According to the scale above the nomogram corresponding to each risk factor, the single score of each factor was obtained, and the total score was obtained by adding the individual scores. The total score was compared to the descending scale to obtain the probability of predicting a high WHO/ISUP nuclear grade. (**B**) Receiver operating characteristic curves of the diagnostic performance of the logistic model for distinguishing high- from low-grade ccRCC

In univariate analysis, D_p_____ROI_Low_, D_p_____ROI_____largest_, D_t_____ROI_Low_, *f*____ROI_Low_, *f*____ROI_____largest_, ADC____ROI_Low_, ADC____ROI_____largest_, T2LIA, peritumoral neovascularization, symptom, RBC in urine, serum albumin, aspartate aminotransferase, alanine aminotransferase, creatine kinase, serum urea, hemoglobin, RBC count, lymphocyte count, platelet count, and tumor size were potentially related to high WHO/ISUP nuclear grade (all *P* < 0.05, Table S4). To adjust for potential confounders, we further explored the potential predictors using multivariable logistic regression analysis (backward LR). D_p_____ROI_Low_, tumor size, serum albumin, platelet count, and lymphocyte count were independently related to high WHO/ISUP nuclear grade (Table S5), and a nomogram was constructed for the multivariable logistic model (Fig. 5A).

Through the multivariable logistic model, the final computer based on five independent features was trained and then applied to the test set. The equation of the computer was as follows:

$$\begin{aligned}Y &= 0.936 + (-1.418) \times X_{1} + 1.334 \times X_{2} + (-1.585) \cr&\quad\times X_{3} + (-1.198) \times X_{4} + 1.631 \times X_{5}\end{aligned}$$

Where X_1_, X_2_, X_3_, X_4_, and X_5_ are equal to 1 when D_p_____ROI_Low_, tumor size, serum albumin, platelet count, and lymphocyte count are above their respective threshold (obtained through the maximum Youden index aforementioned), otherwise equal to 0.

### Diagnostic performance of the multivariable logistic model and the progression-free survival of the patients

The diagnostic performance of the five independent predictors and the logistic model is shown in Table 4. The diagnostic efficacy of independent predictors was moderate (AUC ranged from 0.602 to 0.666). The logistic model combining individual factors presented a better diagnostic performance than each factor (DeLong’s test, 0.0006 ≤ *P* ≤ 0.0054). Figure 5B shows the ROC curves of the model in identifying high WHO/ISUP nuclear grades in the training and test sets. The model demonstrated a good performance in identifying high WHO/ISUP nuclear grade in the training set, with an AUC of 0.817 (95% confidence interval [CI]: 0.735–0.899), a sensitivity of 70.0%, and a specificity of 77.8%. In the independent test set, the AUC of the logistic model was 0.766 (95% CI, 0.567–0.966), the sensitivity was 79.0%, and the specificity was 75.0%.

**Table 4:** The diagnostic performance of independent predictors and logistic regression model in predicting ISUP nuclear grade of clear cell renal cell carcinoma and tumor thrombus

Parameters	AUC	SEN (%)	SPE (%)	PPV (%)	NPV (%)	Threshold	*P* value
Training set							
D_p_____ROI_Low_	0.602 (0.493–0.711)	50.6 (28.9–53.1)	83.3 (67.2–93.6)	82.4 (68.1–91.1)	42.3 (36.4–48.3)	13.677 × 10^− 3^ mm^2^/s	0.066
Tumor size	0.618 (0.501–0.735)	72.5 (60.4–82.5)	50.0 (32.9–67.1)	73.5 (66.0-79.9)	48.6 (36.4–61.0)	6.7 cm	0.048
Lymphocyte count	0.617 (0.504–0.729)	47.8 (35.6–60.2)	80.6 (64.0-91.8)	82.5 (69.9–90.5)	44.6 (37.9–51.5)	1.388 × 10^9^/L	0.042
Platelet count	0.666 (0.553–0.778)	87.0 (76.7–93.9)	41.7 (25.5–59.2)	74.1 (68.1–79.3)	62.5 (44.8–77.4)	178 × 10^9^/L	0.004
Serum albumin	0.633 (0.515–0.752)	84.1 (73.3–91.8)	47.2 (30.4–64.5)	75.3 (68.8–80.9)	60.7 (44.8–74.6)	42.4 g/L	0.028
Logistic model	0.817 (0.735–0.899)	70.0 (57.3–80.1)	77.8 (60.8–89.9)	85.7 (76.2–91.9)	57.1 (47.3–66.5)	1.12	< 0.001
Test set							
D_p_____ROI_Low_	0.605 (0.351–0.860)	94.7 (74.0-99.9)	37.5 (8.5–75.5)	78.3 (67.6–86.2)	75.0 (26.7–96.1)	13.677 × 10^− 3^ mm^2^/s	0.418
Tumor size	0.671 (0.426–0.916)	73.7 (48.8–90.9)	62.5 (24.5–91.5)	82.4 (64.7–92.2)	50.0 (28.4–71.6)	6.7 cm	0.172
Lymphocyte count	0.618 (0.366–0.871)	68.4 (43.4–87.4)	75.0 (34.9–96.8)	86.7 (65.3–95.7)	50.0 (31.6–68.4)	1.388 × 10^9^/L	0.358
Platelet count	0.609 (0.378–0.839)	36.8 (16.3–61.6)	87.5 (47.3–99.7)	87.5 (50.5–98.0)	36.8 (27.5–47.3)	178 × 10^9^/L	0.356
Serum albumin	0.523 (0.291–0.755)	42.1 (20.3–66.5)	87.5 (47.3–99.7)	88.9 (54.3–98.2)	38.9 (28.6–50.3)	42.4 g/L	0.846
Logistic model	0.766 (0.567–0.966)	79.0 (54.4–93.9)	75.0 (34.9–96.8)	88.2 (68.8–96.2)	60.0 (36.5–79.6)	1.12	0.009

Note The data shown in brackets represent the 95% confidence intervals (CIs). AUC = area under the curve; SEN = sensitivity; SPE = specificity; PPV = positive predict value; NPV = negative predict value

**Fig. 6:**
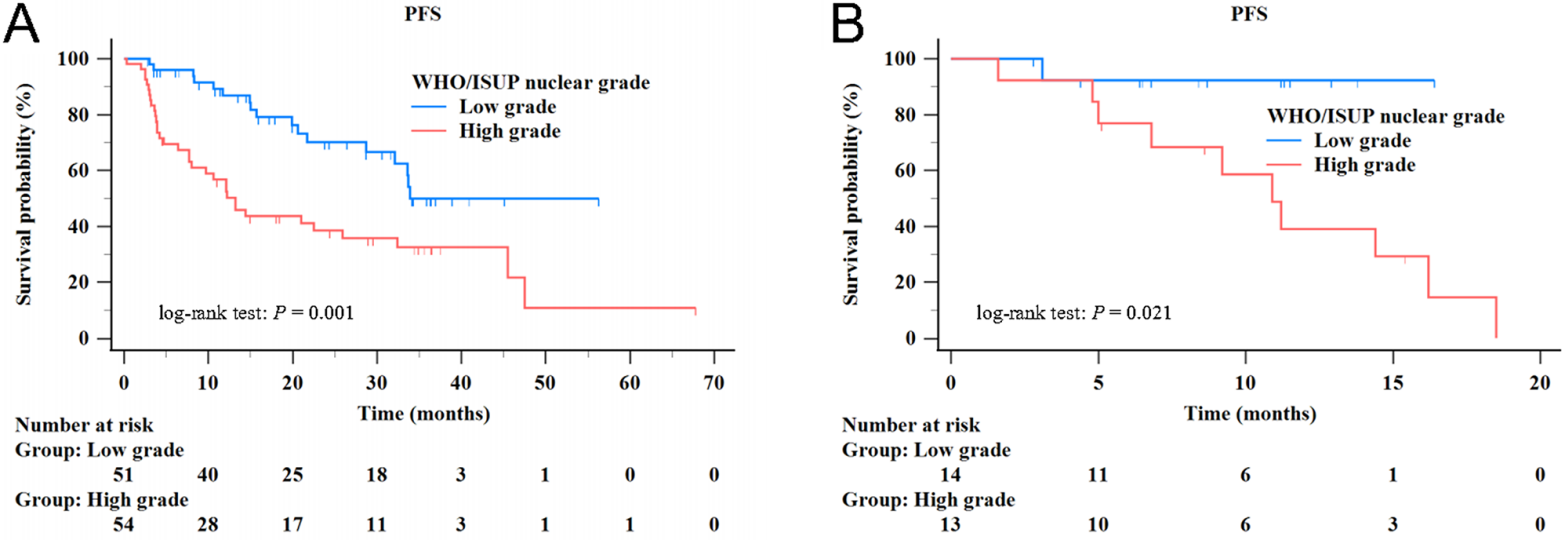
Kaplan-Meier plots comparing progression-free survival of patients with predicted low and high WHO/ISUP nuclear grade clear cell renal cell carcinoma complicated with venous tumor thrombus. (**A**) The training set. (**B**) The test set

Kaplan–Meier analysis showed that the predicted high WHO/ISUP nuclear grade group had poorer PFS than the low WHO/ISUP nuclear grade group in both the training and test sets. In the training set, the median PFS of the predicted low and high WHO/ISUP nuclear grade groups were 33.9 months (95% CI: 28.7–33.9 months) and 13.2 months (range, 7.7–25.9 months) (*P* = 0.001, Fig. 6A). In the test set, the median PFS of the predicted low WHO/ISUP nuclear grade group was not reached, whereas the median PFS of the predicted high WHO/ISUP nuclear grade groups was 10.9 months (range, 5.0–16.2 months) (*P* = 0.021, Fig. 6B).

### Calibration verification and clinical application of the nomogram

**Fig. 7:**
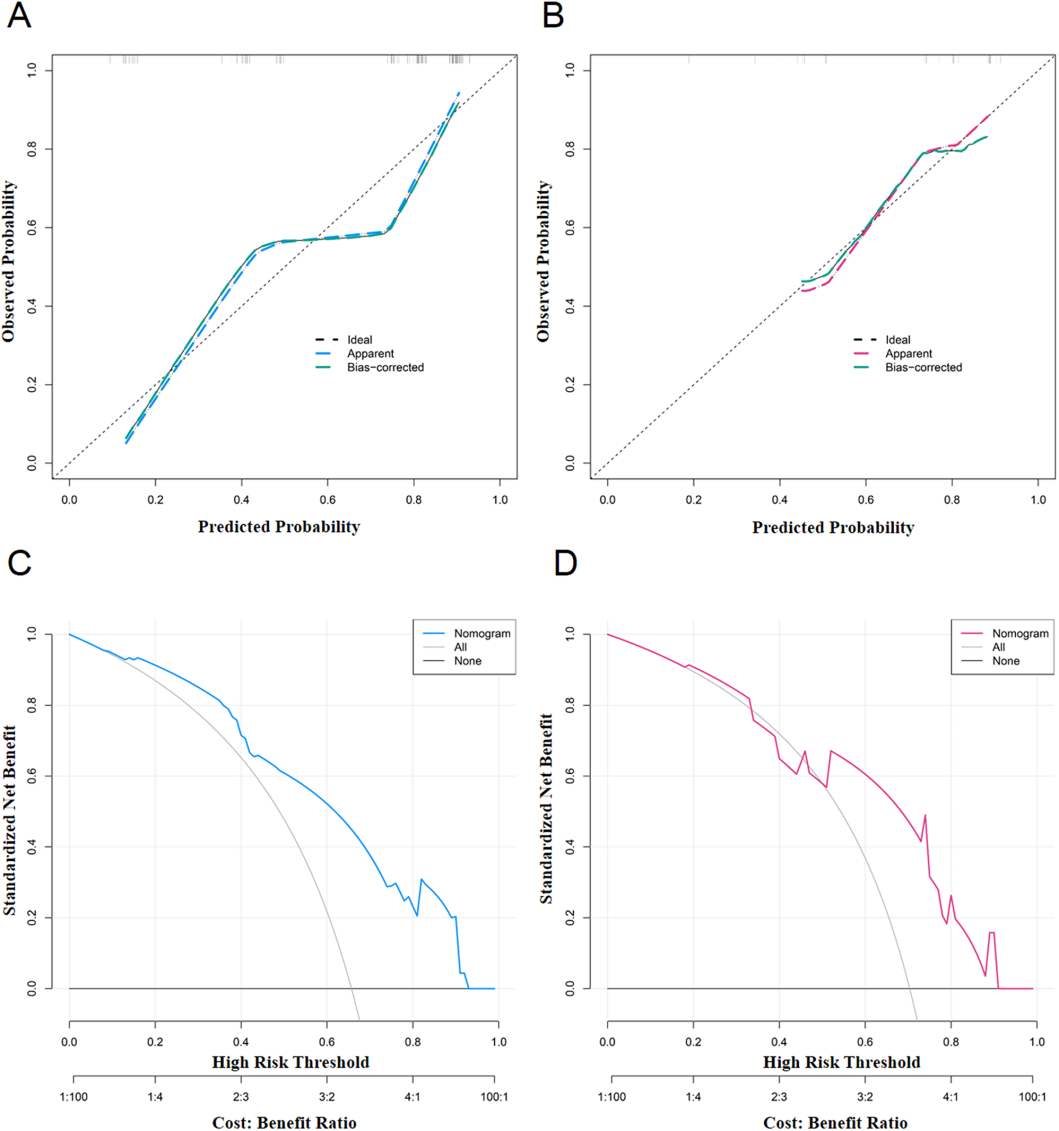
Calibration curve and decision curve analysis of nomogram model for predicting high WHO/ISUP nuclear grade ccRCC. Calibration curve of the training set (**A**) and test set (**B**). A new sample data set was obtained by using R4.1 software through the Bootstraps method of independent sampling 2000 times, and calibration curves of high WHO/ISUP nuclear grade ccRCC occurrence risk line prediction were drawn. The X-axis represents the probability of high WHO/ISUP nuclear grade ccRCC predicted by the nomogram model, and the Y-axis represents the probability of high WHO/ISUP nuclear grade ccRCC being diagnosed actually. Decision curve analysis (DCA) of the training set (**C**) and test set (**D**). The net benefit of the logistic model was demonstrated to be superior to that of treat-all and treat-none protocol

The calibration curves of the nomogram in the training and test set are shown in Fig. 7A and B. The calibration curve shows that good calibration was observed between the predicted result of the nomogram and the real result, and the difference was not significant in the Hosmer-Lemeshow test (χ^2^ = 4.859 and 3.324, *P* = 0.677 and 0.767, respectively). The clinical utility of the nomogram was evaluated by decision curve analysis (DCA), and the net benefit was demonstrated to be superior to the treat-all and treat-none strategy in both the training and test sets (Fig. 7C and D).

### Interreader agreement between two independent radiologists

Subjective imaging signs showed substantial to excellent agreement (Kappa value ranged from 0.809 to 0.923) between the two radiologists, except for necrosis (Kappa value: 0.613), pseudo-capsule (Kappa value: 0.697), and T2LIA (Kappa value: 0.578). For the continuous imaging variables, substantial to excellent agreement was reached in D_p_____ROI_____Low_, D_t_____ROI_____Low_, *f*____ROI_____Low_, *f*____ROI_____largest_, ADC____ROI_____Low_, and ADC____ROI_____largest_ (ICC values ranged from 0.758 to 0.887). However, the ICC value of D_p_____ROI_____largest_ and D_t_____ROI_____largest_ were merely 0.589 and 0.593, indicating a moderate agreement.

## Discussion

Identifying patients with high WHO/ISUP nuclear grades may help clinical decision-making in ccRCC and VTT. Our results showed that IVIM parameters and clinical indicators are of great value in differentiating low and high WHO/ISUP nuclear grades of ccRCC and VTT. In addition, the constructed model can be used to distinguish the prognosis of patients.

MRI can provide multiple anatomical and functional information, and few studies predicted WHO/ISUP grade through MRI [32–35]. Among conventional imaging signs, only tumor size was confirmed as an independent risk factor related to high WHO/ISUP nuclear grade in our study. Consistent with our findings, a previous study reported that mean lesion size was larger in high-grade ccRCC [14]. This phenomenon is not difficult to explain. The larger the tumor grows, the more complex the internal environment of the tumor, and the later the tumor stages. In a previous study, the proportion of high-grade tumors was 0% for tumors < 1 cm, while the proportion of high-grade tumors was 59% for tumors > 7 cm [37].

Magnetic resonance diffusion-weighted imaging has great application prospects in distinguishing WHO/ISUP nuclear grade of renal cell carcinoma. A meta-analysis reviewed the diagnostic value of DWI in distinguishing high-grade and low-grade ccRCC, and pooled sensitivity and specificity were 0.78 and 0.86 [38]. ADC has been reported to distinguish low-grade and high-grade ccRCC [39]. However, ADC was not a true molecular diffusion, it contained some source of incoherent motion. A previous study has shown that IVIM parameters perform better than ADC in distinguishing ccRCC grading [36]. Another study showed that the f-value can more accurately characterize the Fuhrman grading of ccRCC than the ADC value [35]. In our study, ADC was potentially related to high WHO/ISUP nuclear grade in univariate analysis but failed to remain an independent predictor. Instead, we found that D_p_ROI_Low_ was independently correlated with high WHO/ISUP nuclear grade. D_p_ was an index reflecting capillary vascular perfusion [40]. Lower D_p_ values in the High WHO/ISUP nuclear grade group indicate that the blood perfusion in these tumors was less, and the tumor microenvironment was hypoxia. Previous studies also demonstrated that D_p_ values correlated negatively with ccRCC grading [33, 34]. However, specific studies targeting ccRCC complicated with VTT are lacking. We found that parameters from IVIM-DWI could be used to predict the WHO/ISUP grade of ccRCC complicated with VTT. However, the diagnostic performance of a single indicator is poor, and it is necessary to combine multiple indicators to improve diagnostic accuracy.

We further explored the value of preoperative clinical and laboratory indicators in predicting the WHO/ISUP grade of ccRCC complicated with VTT, and we found that serum albumin, platelet count, and lymphocyte count were independently correlated with the high WHO/ISUP nuclear grade of ccRCC and VTT. In a previous study, the neutrophil-to-lymphocyte and platelet-to-lymphocyte ratios were significantly higher in patients with high Fuhrman grade RCC [41]. We speculate that the WHO/ISUP nuclear grade of RCC is related to the tumor microenvironment. Inflammation plays an important role in the onset and progression of RCC, with lymphocytes and platelets playing prominent roles in tumor inflammation and immunology [42]. Lymphocytes play a crucial role in the lymphocyte-mediated immune response to tumors. Platelets can release vascular endothelial growth factor, which plays an important role in tumor angiogenesis [41]. Systemic immune response score, defined as platelet × neutrophil-to-lymphocyte ratio, was found to be a significant marker of prostate cancer with higher ISUP grades [43]. The serum albumin level could be used to assess nutritional conditions [44]. In our study, hypoalbuminemia was more frequent in patients with high WHO/ISUP nuclear grade, reflecting insufficient nutrition. We speculate that the higher the WHO/ISUP nuclear grade, the more nutrients consumed, leading to low albumin levels. These findings imply the reason for the poorer prognosis of patients with high WHO/ISUP nuclear grades.

Artificial intelligence is currently a hot field. Texture analysis based on apparent diffusion coefficient maps has been reported for distinguishing chromophobe renal cell carcinoma from renal oncocytoma [45], and fat-poor angiomyolipoma from non-clear-cell renal cell carcinoma [46]. Another study used radiomics features based on diffusion-weighted magnetic resonance imaging to distinguish between fat-poor angiomyolipoma and ccRCC [47]. These studies have achieved satisfactory results. CT-based radiomics models were also reported to distinguish low-grade and high-grade ccRCC [15, 47–51]. However, those studies only included a small population, and an independent validation group was often absent. In the future, we will use artificial intelligence methods to predict the WHO/ISUP nuclear grade of ccRCC and VTT.

We acknowledge that our research has some limitations. First, this study was conducted in a single center and the images were acquired on one MR scanner. External validation is required to confirm our results. Secondly, the sample size in this study was relatively small. However, we focused on RCC complicated with tumor thrombus, which was relatively rare but was one of the most challenging diseases in urology. We will collect more cases for further research. Thirdly, the research design method we used to construct the model through the retrospective collection of cases may have selection bias. Fourthly, we focused on renal cell carcinoma with concomitant venous tumor thrombus and surgical resection, so this model may not be applicable to patients without concomitant tumor thrombus. In addition, tumors with tumor thrombus may be more invasive, resulting in a higher proportion of high nuclear grades, which may also affect selection bias. Lastly, the respiratory movement of the kidneys mainly occurs in a craniocaudal direction and does not always coincide with the movement of the abdominal wall, which may introduce bias in measurement.

### Summary

In conclusion, IVIM-DWI-derived parameters and clinical indicators can be used to differentiate nuclear grades and predict progression-free survival of ccRCC and VTT. Accurately preoperative identification of patients with high WHO/ISUP grade ccRCC and VTT may help in surgical program selection and prognosis assessment. When encountering patients with ccRCC and VTT, this model can be used to predict the nuclear grading of tumors non-invasively. It is recommended that patients with high nuclear-grade tumors receive neoadjuvant therapy, ultimately improving their prognosis.

## Electronic Supplementary Material

Below is the link to the electronic supplementary material.

Supplementary Material 1

## Data Availability

No datasets were generated or analysed during the current study.

## Abbreviations

AUC: Area under the ROC curve
ADC: Apparent diffusion coefficient
BMI: Body mass index
DCA: Decision curve analysis
IVIM-DWI: Intravoxel incoherent motion diffusion-weighted imaging
IQR: Interquartile range
ICC: Intraclass correlation coefficient
NEX: Number of excitations
OR: Odds ratio
PFS: Progression-free survival
RBC: Red blood cell
RCC: Renal cell carcinoma
ROI: Regions of interest
ROC: Receiver operating characteristic
SD: Standard deviation
T2LIA: T2 low-intensity areas
VTT: Venous tumor thrombus
WHO/ISUP: World Health Organization and International Society of Urological Pathology
WBC: White blood cell
