# Supplementary material for: Development and validation of intravoxel incoherent motion diffusion weighted imaging-based model for preoperative distinguishing nuclear grade and survival of clear cell renal cell carcinoma complicated with venous tumor thrombus
Source: Cancer Imaging. 2024 Dec 18;24:164. doi: 10.1186/s40644-024-00816-2 (PMC11654007; doi:10.1186/s40644-024-00816-2)
Supplement: Supplementary file 1 — Supplementary Material 1 [file 40644_2024_816_MOESM1_ESM.docx]

FOV, field of view; FSE, fast spin echo; SSFSE, single shot fast spin echo; LAVA, liver acquisition with volume acceleration

| **Table S1** MRI scanner, scanning sequences and parameters | | | | | | | | |
| --- | --- | --- | --- | --- | --- | --- | --- | --- |
| **MRI Scanner** | **Filed strength** | **Sequence** | **Section thickness/gap (mm)** | **TR / TE (ms)** | **FOV (cm×cm)** | **Bandwidth (Hz)** | **Matrix** | **Average** |
| GE Discovery MR750 | 3.0T | Axial FSE T2WI | 5.0/0.5 | 8000~12000/110 | 38 × 40 | 50 | 320 × 320 | 1.5 |
|  |  | Coronal SSFSE T2WI | 5.0/0.5 | 6000~8000/110 | 38 × 40 | 62.5 | 288 × 224 | 1 |
|  |  | In-and-out-of-phase T1-weighted spoiled gradient- recalled dual-echo sequence | 4.0/0.4 | 3.7/1.1 | 38 × 40 | 166.67 | 256 × 256 | 1 |
|  |  | Axial T1 WI LAVA | 5.0/-2.0 | 3.9/1.7 | 36 × 40 | 200 | 320 × 224 | 1 |
|  |  | Axial T1 dynamic contrast enhancement LAVA | 5.0/-2.0 | 3.9/1.3 | 36 × 40 | 125 | 288 × 192 | 1 |
|  |  | Axial single-shot echo planar imaging sequence | 5.0/1.0 | 3500/58 | 38 × 38 | 110 | 160 × 160 | 2 |

| **Table S2** The maximum Youden index and related parameters corresponding to each continuous variable | | | | | | |
| --- | --- | --- | --- | --- | --- | --- |
| **Variable** | **Youden index** | **Associated criterion** | **Sensitivity** | **Specificity** | **AUC(95%CI)** | ***P* value** |
| Alkaline phosphatase | 0.208 | 104.1 U/L | 31.9(21.2-44.2) | 88.9(73.9-96.9) | 0.614(0.502-0.726) | 0.045 |
| Serum urea | 0.309 | 4.5 mmol/L | 42.1(30.2-54.5) | 88.8(73.9-96.9) | 0.624(0.514-0.735) | 0.028 |
| Hemoglobin | 0.374 | 121 g/L | 65.2(52.8-76.3) | 72.2(54.8-85.8) | 0.718(0.612-0.824) | <0.001 |
| Serum albumin | 0.313 | 42.6 g/L | 84.1(73.3-91.8) | 47.2(30.4-64.5) | 0.633(0.515-0.752) | 0.028 |
| Aspartate aminotransferase | 0.299 | 13.6 U/L | 49.3(37.0-61.6) | 80.6(64.0-91.8) | 0.643(0.527-0.758) | 0.016 |
| Alanine aminotransferase | 0.262 | 17.7 U/L | 69.6(57.3-80.1) | 56.6(38.1-72.1) | 0.644(0.529-0.759) | 0.014 |
| Creatine kinase | 0.324 | 42 U/L | 56.5(44.0-68.4) | 75.9(57.8-87.9) | 0.69 (0.588-0.796) | <0.001 |
| RBC count | 0.227 | 4.57×10^12^/L | 78.3(66.7-87.3) | 44.4(27.9-61.9) | 0.623(0.509-0.737) | 0.034 |
| Lymphocyte count | 0.284 | 1.39×10^9^/L | 47.8(35.6-60.2) | 80.6(64.0-91.8) | 0.617(0.504-0.729) | 0.042 |
| Platelet count | 0.286 | 178×10^9^/L | 87.0(76.7-93.9) | 41.6(25.5-59.2) | 0.666(0.553-0.778) | 0.004 |
| Dp_ROI_Low | 0.339 | 13.677×10^-3^ mm^2^/s | 50.6(28.9-53.1) | 83.3(67.2-93.6) | 0.602(0.493-0.711) | 0.066 |
| Dp_ROI_largest | 0.234 | 22.598×10^-3^ mm^2^/s | 62.3(49.8-73.7) | 61.1(43.5-76.9) | 0.592(0.477-0.707) | 0.117 |
| Dt_ROI_Low | 0.236 | 0.925×10^-3^ mm^2^/s | 65.2(52.8-76.3) | 58.4(40.8-74.5) | 0.609(0.485-0.733) | 0.086 |
| Dt_ ROI_largest | 0.217 | 1.287×10^-3^ mm^2^/s | 55.1(42.6-67.1) | 66.6(49.0-81.4) | 0.613(0.498-0.728) | 0.054 |
| f_ROI_Low | 0.245 | 0.262 | 55.1(39.8-64.4) | 69.4(51.9-83.7) | 0.641(0.526-0.756) | 0.016 |
| f_ROI_largest | 0.216 | 0.382 | 85.5(75.0-92.8) | 36.1(20.8-53.8) | 0.583(0.459-0.706) | 0.191 |
| ADC_ROI_Low | 0.405 | 1.135×10^-3^ mm^2^/s | 91.3(82.0-96.7) | 49.2(20.8-53.8) | 0.716(0.601-0.832) | <0.001 |
| ADC_ROI_largest | 0.297 | 1.824×10^-3^ mm^2^/s | 79.7(68.3-88.4) | 50.0(32.9-67.1) | 0.631(0.511-0.752) | 0.032 |
| Tumor size | 0.225 | 6.7 cm | 72.5(60.4-82.5) | 50.0 (32.9-67.1) | 0.618(0.501-0.735) | 0.048 |

| **Table S3** Comparison of clinical features between patients with low and high ISUP nuclear grade clear cell renal cell carcinoma in the training set | | | |
| --- | --- | --- | --- |
| **Parameter** | **Low-grade (n = 36)** | **High-grade (n = 69)** | ***P* value** |
| BMI |  |  | 0.062 |
| <22.8 kg/m^2^ | 5 (13.9) | 21 (30.4) |  |
| ≥22.8 kg/m^2^ | 31 (86.1) | 48 (69.6) |  |
| Diabetes |  |  | 0.918 |
| Without | 29 (80.6) | 55 (79.7) |  |
| With | 7 (19.4) | 14 (20.3) |  |
| Hypertension |  |  | 0.144 |
| Without | 16 (44.4) | 41 (59.4) |  |
| With | 20 (55.6) | 28 (40.6) |  |
| Smoking history |  |  | 0.063 |
| Without | 14 (38.9) | 40 (58.0) |  |
| With | 22 (61.1) | 29 (42.0) |  |
| Drinking history |  |  | 0.733 |
| Without | 17 (47.2) | 35 (50.7) |  |
| With | 19 (52.8) | 34 (49.3) |  |
| WBC in urine |  |  | 0.925 |
| Negative | 30 (83.3) | 57 (82.6) |  |
| Positive | 6 (16.7) | 12 (17.4) |  |
| Proteinuria |  |  | 0.660 |
| Negative | 24 (66.7) | 43 (62.3) |  |
| Positive | 12 (33.3) | 26 (37.7) |  |
| Total protein |  |  | 0.775 |
| <65.1 g/L | 4 (11.1) | 9 (13.0) |  |
| ≥65.1 g/L | 32 (88.9) | 60 (87.0) |  |
| WBC count |  |  | 0.264 |
| <5.13×10^9^/L | 12 (33.3) | 16 (23.2) |  |
| ≥5.13×10^9^/L | 24 (66.7) | 53 (76.8) |  |
| Neutrophil count |  |  | 0.217 |
| <3.28×10^9^/L | 13 (36.1) | 17 (24.6) |  |
| ≥3.28×10^9^/L | 23 (63.9) | 52 (75.4) |  |
| Monocyte count |  |  | 0.319 |
| <0.39×10^9^/L | 15 (41.7) | 22 (31.9) |  |
| ≥0.39×10^9^/L | 21 (58.3) | 47 (68.1) |  |
| Gamma glutamyltransferase |  |  | 0.313 |
| <33.1 U/L | 23 (63.9) | 37 (53.6) |  |
| ≥33.1 U/L | 13 (36.1) | 32 (46.4) |  |
| Lactate dehydrogenase |  |  | 0.263 |
| <137.7 U/L | 16 (44.4) | 23 (33.3) |  |
| ≥137.7 U/L | 20 (55.6) | 46 (66.7) |  |
| Blood glucose |  |  | 0.344 |
| < 4.6 mmol/L | 12 (33.3) | 17 (24.6) |  |
| ≥ 4.6 mmol/L | 24 (66.7) | 52 (75.4) |  |
| Serum creatinine |  |  | 0.195 |
| < 75.4 μmol/L | 10 (27.8) | 28 (40.6) |  |
| ≥ 75.4 μmol/L | 26 (72.2) | 41 (59.4) |  |
| Serum calcium |  |  | 0.502 |
| < 2.4 mmol/L | 32 (88.9) | 58 (84.1) |  |
| ≥ 2.4 mmol/L | 4 (11.1) | 11 (15.9) |  |
| Note.—Data are numbers of patients, with percentages in parentheses. | | | |

| **Table S4** Univariable logistic regression analysis in predicting ISUP nuclear grade of clear cell renal cell carcinoma and tumor thrombus | | |
| --- | --- | --- |
| **Parameters** | **Odds Ratio (95%CI)** | ***P* value** |
| Age  ≥ 60 years vs. < 60 years | 0.967 (0.431-2.167) | 0.934 |
| Gender  Male vs. Female | 1.312 (0.527-3.268) | 0.559 |
| Side  Right vs. Left | 0.559 (0.245-1.274) | 0.166 |
| Symptom  With vs. Without | 3.393 (1.367-8.422) | 0.008 |
| Diabetes  With vs. Without | 1.055 (0.383-2.903) | 0.918 |
| Hypertension  With vs. Without | 0.546 (0.242-1.233) | 0.146 |
| Smoking history  With vs. Without | 0.461 (0.203-1.051) | 0.065 |
| Drinking history  With vs. Without | 0.869 (0.388-1.948) | 0.733 |
| BMI  ≥ 22.8 kg/m^2^ vs. < 22.8 kg/m^2^ | 0.369 (0.126-1.080) | 0.069 |
| RBC in urine  Positive vs. Negative | 2.300 (1.003-5.275) | 0.049 |
| WBC in urine  Positive vs. Negative | 1.053 (0.359-3.084) | 0.925 |
| Proteinuria  Positive vs. Negative | 1.209 (0.518-2.821) | 0.660 |
| Total protein  ≥ 65.1 g/L vs. < 65.1 g/L | 0.833 (0.238-2.919) | 0.776 |
| Serum albumin  ≥ 42.6 g/L vs. < 42.6 g/L | 0.212 (0.085-0.531) | 0.001 |
| Gamma glutamyltransferase  ≥ 33.1 U/L vs. < 33.1 U/L | 1.530 (0.668-3.504) | 0.314 |
| Lactate dehydrogenase  ≥ 137.7 U/L vs. < 137.7 U/L | 1.600 (0.700-3.656) | 0.265 |
| Aspartate aminotransferase  ≥ 13.6 U/L vs. < 13.6 U/L | 0.296 (0.114-0.767) | 0.012 |
| Alanine aminotransferase  ≥ 17.7 U/L vs. < 17.7 U/L | 0.374 (0.163-0.859) | 0.020 |
| Creatine kinase  ≥ 42 U/L vs. < 42 U/L | 0.272 (0.112-0.663) | 0.004 |
| Alkaline phosphatase  ≥ 104.1 U/L vs. < 104.1 U/L | 2.902 (0.994-8.475) | 0.051 |
| Blood glucose  ≥ 4.6 mmol/L vs. < 4.6 mmol/L | 1.529 (0.632-3.699) | 0.346 |
| Serum urea  ≥ 4.52 mmol/L vs. < 4.52 mmol/L | 0.183 (0.058-0.575) | 0.004 |
| Serum creatinine  ≥ 75.4 μmol/L vs < 75.4 μmol/L | 0.563 (0.235-1.349) | 0.198 |
| Serum calcium  ≥ 2.4 mmol/L vs. < 2.4 mmol/L | 1.517 (0.447-5.155) | 0.504 |
| Hemoglobin  ≥ 121 g/L vs. < 121 g/L | 0.214 (0.087-0.525) | 0.001 |
| RBC count  ≥ 4.57×10^12^/L vs. < 4.57×10^12^/L | 0.347 (0.145-0.830) | 0.017 |
| WBC count  ≥ 5.13×10^9^/L vs. < 5.13×10^9^/L | 1.656 (0.680-4.035) | 0.267 |
| Neutrophil count  ≥ 3.28×10^9^/L vs. < 3.28×10^9^/L | 1.729 (0.722-4.140) | 0.219 |
| Lymphocyte count  ≥ 1.39×10^9^/L vs. < 1.39×10^9^/L | 0.279 (0.108-0.723) | 0.009 |
| Monocyte count  ≥ 0.39×10^9^/L vs. < 0.39×10^9^/L | 1.526 (0.663-3.513) | 0.321 |
| Platelet count  ≥ 178×10^9^/L vs. < 178×10^9^/L | 4.762 (1.815-12.490) | 0.002 |
| Tumor size  ≥ 6.7 cm vs. < 6.7 cm | 2.355 (1.016-5.459) | 0.046 |
| Dp__ROI_Low_  ≥ 13.677×10^-3^ mm^2^/s vs. < 13.677×10^-3^ mm^2^/s | 0.284 (0.098-0.823) | 0.020 |
| Dp_ _ROI___largest_  ≥ 22.598×10^-3^ mm^2^/s vs. < 22.598×10^-3^ mm^2^/s | 0.409 (0.179-0.935) | 0.034 |
| Dt__ROI_Low_  ≥ 0.925×10^-3^ mm^2^/s vs. < 0.925×10^-3^ mm^2^/s | 0.353(0.151-0.822) | 0.016 |
| Dt_ _ROI___largest_  ≥ 1.287×10^-3^ mm^2^/s vs. < 1.287×10^-3^ mm^2^/s | 0.432 (0.187-1.001) | 0.050 |
| *f*__ROI_Low_  ≥ 0.262 vs. < 0.262 | 0.381 (0.162-0.893) | 0.026 |
| *f*_ _ROI___largest_  ≥ 0.382 vs. < 0.382 | 0.394 (0.169-0.920) | 0.031 |
| ADC__ROI_Low_  ≥ 1.135×10^-3^ mm^2^/s vs. < 1.135×10^-3^ mm^2^/s | 0.180 (0.075-0.433) | <0.001 |
| ADC_ _ROI___largest_  ≥ 1.824×10^-3^ mm^2^/s vs. < 1.824×10^-3^ mm^2^/s | 0.278 (0.117-0.662) | 0.004 |
| Infiltrative growth  With vs. Without | 1.618 (0.713-3.671) | 0.250 |
| Pseudocapsule  With vs. Without | 0.906 (0.402-2.043) | 0.812 |
| Hypo-intensity on T_2_WI  With vs. Without | 2.450 (1.063-5.647) | 0.035 |
| Perirenal fat invasion  With vs. Without | 2.067 (0.912-4.685) | 0.082 |
| Peritumoral neovascularization  With vs. Without | 2.636 (1.023-6.792) | 0.045 |
| Intratumoral hemorrhage  With vs. Without | 1.208 (0.525-2.778) | 0.656 |
| Intratumoral necrosis  With vs. Without | 2.366 (0.994-5.630) | 0.052 |
| Note— The cut-off values of continuous variables were determined by the maximized value of the Youden index. BMI = body mass index, RBC = red blood cell, WBC = white blood cell, DWI = diffusion weighted imaging, ADC = apparent diffusion coefficient. T2LIA= Hypo-intensity on T_2_WI | | |

| **Table S5** Multivariable logistic regression analysis for predicting ISUP nuclear grade of clear cell renal cell carcinoma complicated with tumor thrombus in the training tet | | |
| --- | --- | --- |
| **Variable** | **Odds Ratio (95% CI)** | ***P* value** |
| D_p___ROI_Low_  ≥14.038×10^-3^ mm^2^/s vs < 14.038×10^-3^ mm^2^/s | 0.242 (0.070-0.838) | 0.025 |
| Tumor size  ≥ 6.7 cm vs < 6.7 cm | 3.797 (1.286-11.212) | 0.016 |
| Serum albumin  ≥ 42.6 g/L vs < 42.6 g/L | 0.205 (0.066-0.639) | 0.006 |
| Platelet count  ≥ 178×10^9^/L vs < 178×10^9^/L | 0.302 (0.102-0.893) | 0.030 |
| Lymphocyte count  ≥1.388×10^9^/L vs < 1.388××10^9^/L | 5.110 (1.586-16.467) | 0.006 |
| Note—The cut-off values were determined by the maximized value of the Youden index. | | |

#### Calculation code

%% General settings

dataFolder = {'E:\IVIM-Processed'};

def = {'20 50 100 150 200 400 600 800 0','200'};

%def = {'10 20 30 40 50 100 200 300 500 1000 1500 2000 3000 0','200'}; %ZHFU

bCutOff = str2double(def{2});

filterSigma = 0.6;

filterSize = 5; % isotropic gaussian filter

fitOptions = optimset('MaxFunEvals',10000,'MaxIter',10000,'TolFun',1e-6);

if ispc

slash = '\';

else

slash = '/';

end

%% Calculation

eval(sprintf('cd %s',dataFolder{1}));

subjList = pywu_GetFileList;

for subj = 1:length(subjList)

b = str2num(def{1})'; %#ok<*ST2NM>

eval(sprintf('cd %s%s%s',dataFolder{1},slash,subjList{subj}));

fileList = pywu_GetFileList;

fileList(strcmp('data_StandardADC',fileList)) = [];

fileList(strcmp('data_f',fileList)) = [];

fileList(strcmp('data_Dslow',fileList)) = [];

fileList(strcmp('data_Dfast',fileList)) = [];

fileList(strcmp('data_MD',fileList)) = [];

fileList(strcmp('data_MK',fileList)) = [];

imgNum = length(fileList);

sliceNum = imgNum/length(b);

data = zeros(256,256,imgNum);

insNum = zeros(imgNum,1);

for s = 1:imgNum

img = imresize(double(dicomread(fileList{s})),[256 256],'bilinear');

data(:,:,s) = imgaussfilt(img,filterSigma,'FilterSize',filterSize); % gaussian filtering

hdr = dicominfo(fileList{s});

insNum(s) = hdr.InstanceNumber;

end

data(data<0) = 0;

[~,idx1] = sort(insNum);

data = reshape(data(:,:,idx1),256,256,sliceNum,length(b));

[b,idx2] = sort(b);

data = data(:,:,:,idx2);

bHigh = b(b>=bCutOff);

dataHigh = data(:,:,:,b>=bCutOff);

thresholdL = 100;

mask = imbinarize(data(:,:,:,1),thresholdL);

ADC = zeros(256,256,sliceNum);

f = zeros(256,256,sliceNum);

D = zeros(256,256,sliceNum);

Dfast = zeros(256,256,sliceNum);

MD = zeros(256,256,sliceNum);

MK = zeros(256,256,sliceNum);

X1 = ones(length(b),1);

X1(:,2) = b;

X1_prep = pinv(X1'*X1)*X1';

X2 = ones(length(bHigh),1);

X2(:,2) = bHigh;

X2_prep = pinv(X2'*X2)*X2';

[idx_r,idx_c,idx_s] = ind2sub([256,256,sliceNum],find(mask==1));

h = waitbar(0,'IVIM and IVIM-DKI fitting in progress...');

for vox = 1:length(idx_r)

dataV = squeeze(data(idx_r(vox),idx_c(vox),idx_s(vox),:));

tmp = X1_prep*log(dataV);

tmp_ADC = -tmp(2);

tmp_ADC(tmp_ADC<0) = 0; tmp_ADC(tmp_ADC>0.005) = 0.005;

ADC(idx_r(vox),idx_c(vox),idx_s(vox)) = tmp_ADC;

dataHighV = squeeze(dataHigh(idx_r(vox),idx_c(vox),idx_s(vox),:));

tmp = X2_prep*log(dataHighV);

tmp_f = 1-exp(tmp(1))/dataV(1);

tmp_f(tmp_f<0) = 0; tmp_f(tmp_f>1) = 1;

tmp_D = -tmp(2);

tmp_D(tmp_D<0) = 0; tmp_D(tmp_D>0.005) = 0.005;

D(idx_r(vox),idx_c(vox),idx_s(vox)) = tmp_D;

params0 = [dataV(1),tmp_f,0.005];

LB = [0,0,0];

UB = [2*dataV(1),1,0.05];

fit_params = fminsearchbnd(@(params)sum((dataV-params(1)*((1-params(2))*exp(-b*tmp_D)+params(2)*exp(-b*params(3)))).^2),params0,LB,UB,fitOptions);

f(idx_r(vox),idx_c(vox),idx_s(vox)) = fit_params(2);

Dfast(idx_r(vox),idx_c(vox),idx_s(vox)) = fit_params(3);

params0 = [dataV(1),0.001,1];

LB = [0,0,0];

UB = [2*dataV(1),0.005,6];

fit_params = fminsearchbnd(@(params)sum((dataHighV-params(1)*exp(-bHigh*params(2)+bHigh.^2*params(2).^2*params(3)/6)).^2),params0,LB,UB,fitOptions);

MD(idx_r(vox),idx_c(vox),idx_s(vox)) = fit_params(2);

MK(idx_r(vox),idx_c(vox),idx_s(vox)) = fit_params(3);

if mod(vox,round(length(idx_r)/100))==0

str = ['IVIM and IVIM-DKI fitting in progress...',num2str(round(vox/length(idx_r)*100)),'%'];

waitbar(vox/length(idx_r),h,str);

end

end

delete(h);

ADCDicom = uint16(ADC*1000000);

fDicom = uint16(f*1000);

DDicom = uint16(D*1000000);

DfastDicom = uint16(Dfast*1000000);

MDDicom = uint16(MD*1000000);

MKDicom = uint16(MK*1000);

mkdir data_StandardADC

cd data_StandardADC

for s = 1:sliceNum

hdr = dicominfo(sprintf('..%s%s',slash,fileList{idx1(imgNum-sliceNum+s)}));

hdr.SeriesDescription = 'Result_StandardADC';

hdr.SeriesNumber = hdr.SeriesNumber*100;

hdr.InstanceNumber = s;

hdr.RescaleIntercept = 0;

hdr.RescaleSlope = 0.001; % in unit of x10^-3 mm^2/s

hdr.WindowCenter = 1;

hdr.WindowWidth = 2;

hdr.IconImageSequence = [];

dicomwrite(squeeze(ADCDicom(:,:,s)),sprintf('StandardADC_%02d.dcm',s),hdr,'CreateMode','Copy');

end

cd ..

mkdir data_f

cd data_f

for s = 1:sliceNum

hdr = dicominfo(sprintf('..%s%s',slash,fileList{idx1(imgNum-sliceNum+s)}));

hdr.SeriesDescription = 'Result_IVIM_f';

hdr.SeriesNumber = hdr.SeriesNumber*100+3;

hdr.InstanceNumber = s;

hdr.RescaleIntercept = 0;

hdr.RescaleSlope = 0.001;

hdr.WindowCenter = 0.5;

hdr.WindowWidth = 1;

hdr.IconImageSequence = [];

dicomwrite(squeeze(fDicom(:,:,s)),sprintf('IVIM_f_%02d.dcm',s),hdr,'CreateMode','Copy');

end

cd ..

mkdir data_Dslow

cd data_Dslow

for s = 1:sliceNum

hdr = dicominfo(sprintf('..%s%s',slash,fileList{idx1(imgNum-sliceNum+s)}));

hdr.SeriesDescription = 'Result_IVIM_D';

hdr.SeriesNumber = hdr.SeriesNumber*100+2;

hdr.InstanceNumber = s;

hdr.RescaleIntercept = 0;

hdr.RescaleSlope = 0.001; % in unit of x10^-3 mm^2/s

hdr.WindowCenter = 1;

hdr.WindowWidth = 2;

hdr.IconImageSequence = [];

dicomwrite(squeeze(DDicom(:,:,s)),sprintf('IVIM_D_%02d.dcm',s),hdr,'CreateMode','Copy');

end

cd ..

mkdir data_Dfast

cd data_Dfast

for s = 1:sliceNum

hdr = dicominfo(sprintf('..%s%s',slash,fileList{idx1(imgNum-sliceNum+s)}));

hdr.SeriesDescription = 'Result_IVIM_Dfast';

hdr.SeriesNumber = hdr.SeriesNumber*100+1;

hdr.InstanceNumber = s;

hdr.RescaleIntercept = 0;

hdr.RescaleSlope = 0.001; % in unit of x10^-3 mm^2/s

hdr.WindowCenter = 5;

hdr.WindowWidth = 10;

hdr.IconImageSequence = [];

dicomwrite(squeeze(DfastDicom(:,:,s)),sprintf('IVIM_Dfast_%02d.dcm',s),hdr,'CreateMode','Copy');

end

**References**

1. Iima M, Le Bihan D. Clinical Intravoxel Incoherent Motion and Diffusion MR Imaging: Past, Present, and Future. Radiology. 2016; 278(1):13-32.

2. Partridge SC, Zhang Z, Newitt DC, et al. Diffusion-weighted MRI Findings Predict Pathologic Response in Neoadjuvant Treatment of Breast Cancer: The ACRIN 6698 Multicenter Trial. Radiology. 2018; 289(3):618-27.
